# A comparative analysis of early child health and development services and outcomes in countries with different redistributive policies

**DOI:** 10.1186/1471-2458-13-1049

**Published:** 2013-11-06

**Authors:** Meta van den Heuvel, Jessica Hopkins, Anne Biscaro, Cinntha Srikanthan, Andrea Feller, Sven Bremberg, Nienke Verkuijl, Boudien Flapper, Elizabeth Lee Ford-Jones, Robin Williams

**Affiliations:** 1 Department of Pediatrics, Hospital for Sick Children and the University of Toronto, Toronto, Ontario, Canada; 2 Department of Social Pediatrics, Hospital for Sick Children and the University of Toronto, Toronto, Ontario, Canada; 3 Department of Pediatrics, Division of Social Pediatrics, University Medical Center Groningen, Groningen, The Netherlands; 4 Niagara Region Public Health, Thorold, Ontario, Canada; 5 Department of Clinical Epidemiology & Biostatistics, McMaster University, Hamilton, Ontario, Canada; 6 McMaster University Medical School, Hamilton, Ontario, Canada; 7 Swedish National Institute of Public Health, Stockholm, Sweden; 8 Department of Public Health, Karolinska Institute, Stockholm, Sweden; 9 Child and Adolescent Mental Health Services, Oxford, UK; 10 Ministry of Health and Long-Term Care, Toronto, Ontario, Canada

**Keywords:** Child development, Child health services, Public policy, Child health, Population health

## Abstract

**Background:**

The social environment is a fundamental determinant of early child development and, in turn, early child development is a determinant of health, well-being, and learning skills across the life course. Redistributive policies aimed at reducing social inequalities, such as a welfare state and labour market policies, have shown a positive association with selected health indicators. In this study, we investigated the influence of redistributive policies specifically on the social environment of early child development in five countries with different political traditions. The objective of this analysis was to highlight similarities and differences in social and health services between the countries and their associations with other health outcomes that can inform better global early child development policies and improve early child health and development.

**Methods:**

Four social determinants of early child development were selected to provide a cross-section of key time periods in a child’s life from prenatal to kindergarten. They included: 1) prenatal care, 2) maternal leave, 3) child health care, and 4) child care and early childhood education. We searched international databases and reports (e.g. Organization for Economic Cooperation and Development, World Bank, and UNICEF) to obtain information about early child development policies, services and outcomes.

**Results:**

Although a comparative analysis cannot claim causation, our analysis suggests that redistributive policies aimed at reducing social inequalities are associated with a positive influence on the social determinants of early child development. Generous redistributive policies are associated with a higher maternal leave allowance and pay and more preventive child healthcare visits. A decreasing trend in infant mortality, low birth weight rate, and under five mortality rate were observed with an increase in redistributive policies. No clear influence of redistributive policies was observed on breastfeeding and immunization rates. In the analysis of child care and early education, the lack of uniform measures of early child development outcomes was apparent.

**Conclusions:**

This paper provides further support for an association between redistributive policies and early child health and development outcomes, along with the organization of early child health and development services.

## Background

Social determinants play a critical role in health from the time of conception, through pregnancy, to the post-natal period, and beyond [1]. The social environment is a fundamental determinant of early child development (ECD) (generally defined as the period from birth to 4 to 6 years of age) and, in turn, ECD is a determinant of health, well-being and learning skills across the balance of the life course.[1] Healthy early childhood development influences obesity and stunting, mental health, heart disease, competence in literacy and numeracy, criminality, and economic participation [2]. Therefore, targeted social and health investments not only benefit individual children, but have the potential to “lift all children up” on a population scale. A strategy of targeted universalism is especially relevant during times of economic restraint given the need to spend public dollars cost-effectively, but also to minimize the “gap” between rich and poor within countries. Interestingly, there is not a clear relationship between child well-being and gross domestic product (GDP) per capita; suggesting that more than a strong economy is required to promote child health [3]. Navarro *et al*. suggest that another factor contributing to child well-being is a country’s redistributive policy [4]. Redistributive policies aimed at reducing social inequalities, such as a welfare state and labour market policies, have been positively associated with selected health indicators, such as infant mortality and life expectancy at birth [4]. Hence, quality, evidence-based ECD policies and services may prove a powerful strategy for rich and poor countries by bridging the gap between economic inputs and child health [5].

In this study, we investigated the influence of redistributive policies on the social environment of ECD in five countries with different political traditions. We selected four social determinants of health important in the early years of development; prenatal care, maternal leave, child health care, and child care and early childhood education (ECE). For each of these social determinants we conducted a comparative analysis of the relevant services and policies with child health and/or development outcomes. The objective of this analysis was to highlight similarities and differences in social and health services between the countries that can inform better global ECD policies and improve early child health and development.

## Methods

### Country selection

Navarro *et al*. have previously described a conceptual framework to identify and study the complex interactions between politics, policy, and public health [4,6,7]. Navarro *et al*.’s method includes identifying countries by political tradition, so that associations between political tradition (as a proxy for policy) and population health outcomes can be made [4,6]. Navarro *et al*. classify governance by political traditions into 4 party types for the period 1950–2000: 1) social democratic parties, 2) Christian democratic or conservative parties in the Judeo-Christian tradition, 3) liberal parties or conservative parties of a liberal persuasion, and 4) conservative dictatorships [4]. Traditionally, social democratic parties have been most committed to redistributive policies. The social policies of these parties have included policies designed to encourage a high proportion of adult men and women to gain employment, generous social transfers and social services, including family-oriented services [4]. Examples of countries with a tradition of social democratic parties include Sweden, Denmark, Finland, and Norway [4]. Christian democratic parties or conservative parties in the Judeo-Christian tradition have been less committed to redistributive policies than the social democrats. They provide social transfers funded mainly by payroll taxes through social security systems. They do not tend to emphasise family-oriented services such as child care [4]. Examples of countries with Christian democratic or conservative Judeo-Christian political traditions include Italy, the Netherlands, west Germany and France [4]. Liberal or conservative parties of a liberal persuasion have not traditionally had a strong commitment to redistributive policies. They do not provide universal social services. Most social services benefits in these countries are means tested, and public social expenditures are much lower than in the countries governed by social democratic and Christian democratic parties [4]. Examples of countries in this tradition include the United Kingdom, Canada, Ireland, and the United States [4]. Lastly, the conservative dictatorships can be characterized by ultra-conservative or authoritarian (fascist) regimes, which can be generally characterized by low social system support and high income inequality. Although not currently dictatorships, examples of historic conservative dictatorships include Spain, Portugal, and Greece [4].

Using the method of Navarro *et al*., [4] we selected five countries representing a cross-section of the political traditions during the time period of 1950 to 2000, including countries governed primarily by: 1) social democratic parties (Sweden), 2) Christian democratic parties or conservative parties in the Judeo-Christian tradition (the Netherlands), 3) liberal parties or conservative parties of a liberal persuasion (Canada, the United States), and 4) conservative dictatorships (Cuba). Although Cuba is not an “ultra-conservative” state and is constitutionally identified as a socialist state, [8] the country is governed an authoritarian regime and is generally recognized as a communist state led for many years by a dictator [9]. These countries were chosen for several reasons. Firstly, Organization for Economic Co-operation and Development (OECD) countries were desired for availability of data. The OECD compiles data and metadata from member countries to allow for member country comparisons. Secondly, in spite of different political traditions and policies, all of these countries have been recognized as global leaders in different aspects of early childhood development (e.g., delivery of prenatal and child health care through polyclinics in Cuba, measurement of school readiness in Canada). Thirdly, trends in early childhood outcomes in countries with different political traditions have been observed in international reports, such as UNICEF’s report on early childhood services [10], suggesting that one need not be exhaustive of all countries to observe trends in early childhood outcomes. Lastly, authors have personal experience working in early child health and development in all of the countries, except Cuba, which allows for a more thorough understanding of contextual factors, which is particularly relevant for analysis of indicators which fall outside those typically collected on international surveys.

### Data sources and indicators

We searched international databases and reports (e.g., OECD, World Bank, and UNICEF) to obtain information on the policies, services, and outcomes of interest. If the information was not available in international databases, we used national or local data sources, as described below.

### Demographic, economic, inequality, and social support

Data pertaining to countries’ demographics and economics (population, population density, immigrants, GDP, tax revenue, and expenditures) were obtained through the OECD’s and World Bank’s databases (OECD.Stat and World Data Bank, respectively) [11,12]. OECD data are obtained from regional- (e.g., Eurostat) or country-level statistical organizations (e.g., Statistics Canada, Statistics Netherlands, Statistics Sweden, National Center for Health Statistics in the United States). Similar to OECD.Stat, the World Data Bank compiles country-level statistical data using information from the statistical systems of member countries. Both databases have made attempts to improve the quality and comparability of data by providing guidance around methodology, sources, and indicator definitions. Data on Cuba are not available in OECD.Stat or the World Data Bank and Cuba does not publicly release statistics for many of the measures of interest. Data on Cuba were obtained from sources in other countries, primarily the United States, which track and monitor indicators of other countries according to standard definitions. For country demographic data, information for Cuba was obtained from the United States Central Intelligence Agency [9].

The Gini coefficient is a measure of income equality with a score of 0 representing perfect equality and 1 perfect inequality [13]. Data for all countries except Cuba were obtained from OECD.Stat [11]. An extensive search for data on Cuba was conducted. No official government source was found, although an article in “The Economist” provided an estimate [14]. This has been included for interest, but should be interpreted with caution as the source, methodology, and definition were not described and may not be comparable to OECD sources.

Like the Gini coefficient, the Inequality-adjusted Human Development Index (IHDI) also measures inequality at the country-level. While the Gini coefficient measures only income inequality, the IHDI measures the level of human development of people in a society while accounting for inequality [15]. It is made up of 3 dimensions, including health, education, and income [15]. A score of 0 represents perfect inequality and a score of 1 perfect equality. IHDI data were obtained from United Nations Human Development reports which use standardized methodologies to draw information from country-level statistical sources for the index calculation [15]. No data were available for Cuba.

Information on health insurance funding was provided by the authors who have personal experience with the included countries.

### Social determinants of early child development

Four social determinants of ECD were selected to provide a cross-section of key time periods in a child’s life from prenatal to kindergarten [1]. They included: 1) prenatal care, 2) maternal leave, 3) child health care, and 4) child care and ECE. For each determinant, relevant policies and services were identified which could influence early child health and development outcomes. Further information on the services and policies of each social determinant and the corresponding outcomes are described below.

### Prenatal care

Prenatal care can provide an effective intervention to improve maternal and child health by supporting positive behaviour changes (e.g., smoking cessation) and connecting parents to prenatal and parenting programs [16,17]. During the intra-partum period, health care providers also have the ability to impact on maternal and infant morbidity and mortality, and promote positive practices, such as breastfeeding. Evidence shows that midwife-led care is associated with an increase in spontaneous vaginal birth and the initiation of breastfeeding [18]. As a policy of interest, we compared the prenatal care services in each country. As outcomes of interest for prenatal care, we identified maternal smoking rate, caesarean-section (c-section) rate, infant mortality rate, and low birth weight rate.

Data for policies and outcomes of prenatal care were generally taken from national statistical databases [19-22]. The c-section rate for Cuba was obtained from the World Health Organization, [20] and data for all countries on infant mortality rate and low birth weight rate (<2500 g) were obtained from the OECD and World Bank [11,12]. Based on standard definitions, data on c-section rate, infant mortality rate, and low birth weight rate have good comparability. Country comparisons for maternal smoking rate and c-section rate were limited by data availability as data were not always available for the same year [23-26]. Where years of data collection differ, this has been noted in the corresponding tables.

### Maternal leave

Maternal leave is recognized as an important component of child health and enabler of breastfeeding and attachment [10]. Breast milk promotes sensory and cognitive development, and protects the infant against infectious and chronic diseases [27,28]. Exclusive breastfeeding reduces infant mortality due to common childhood illnesses, such as diarrhea or pneumonia, and helps for a quicker recovery during illness [27]. These effects can be measured in low and to some extent high income countries [29]. International experts recommend exclusive breastfeeding up to 6 months of age with continued breastfeeding up to 2 years and beyond [30,31]. For policies related to maternal leave, we included maternal leave allowance, maternal leave pay, and parental leave. As a maternal leave outcome, we identified maternal breastfeeding rates (initiation and sustained).

Data for policies on maternal leave (i.e., maternal leave allowance, maternal leave, and parental leave) were obtained primarily from country-level government sources, with the exception of Cuba’s data which were obtained from an academic source [32-36]. Data for maternal leave outcomes were generally taken from statistical databases [11,37]. Information on breastfeeding for Cuba was obtained from UNICEF [38]. Although most countries obtained information on initiation of breastfeeding through routine collection, information on exclusive breastfeeding at 6 months was more difficult to obtain and was taken from a variety of sources, including one-time surveys (Canadian maternity experiences survey, breastfeeding report card in the United States), [19,39] population statistics (Sweden, the Netherlands), [23,37] and UNICEF (Cuba) [38]. Although definitions for exclusive breastfeeding are similar, challenges to comparability arise from different time periods of data collection and methods of sampling populations.

### Child health care

Child health care includes acute and preventive care. Beyond moral and professional obligations, the health sector has a unique opportunity to promote healthy child development because of the high levels of interaction with children and their parents during early childhood [40]. In each country, we compared the acute and preventive child health care services and the number of recommended preventive care visits and approximate cost per child per year [United States dollars (USD)]. Outcomes of interest for child health care and services included coverage of preventive visits, childhood immunization coverage rates, and the under 5 years mortality rate.

Data on policies for child health care were obtained through knowledge of the authors working in their respective countries, and through academic sources for Cuba [41]. Outcome data for service coverage was only available for 2 countries (Sweden and the Netherlands) [42,43]. However, under 5 years mortality and vaccination coverage data were available for all countries through UNICEF [44].

### Child care and early childhood education

Today’s generation of children is the first in which a majority are spending a large part of early childhood in some form of out-of-home child care [45]. Neuroscientific research demonstrates that loving, stable, secure and stimulating relationships with caregivers in the earliest months and years of life are critical for every aspect of a child’s development [45]. We compared the ECE and education services and policies in each country. We focused on the organization of child care, pre-school, and kindergarten and looked specifically at age of enrollment, hours of operation, and contribution from parents and government. As outcomes of ECE and education, we included the Child Development Index (CDI), the Early Development Instrument (EDI), and the educational achievement of 15 year-olds.

Child care centres, or daycares, are places where parents can take children for care while they are otherwise occupied (e.g., while at work). There is generally some cost borne by the parent. Child care centres may be formal institutions or home-based. There are no global databases that collect comprehensive information on child care centres; information was obtained from various reports [42,46-49].

Pre-school or infant education is a formal environment to stimulate child development in multiple domains. Programs may or may not charge parents a fee. Again, no global database containing this information exists, so data were compiled from various reports [50,51].

Kindergarten represents the first formal education level in school. Data on kindergarten start age was obtained from national policy sources.

School readiness measures a child’s preparedness to perform in multiple domains (e.g., emotional, behavioural, cognitive) in the school environment. There are no globally accepted measures of school readiness, however several different methods have been developed and are used to differing degrees around the world [52-55]. For example, the EDI is an internationally recognized tool which can be used to assess school readiness on a population-level [56]. The instrument evaluates children in 5 key domains of early development: physical, social, emotional, communication, and language and cognitive skills. Children who fall in the lowest 10^th^ percentile for a given domain are deemed “vulnerable” in that area [57]. Data in this area are presented for interest, but comparability is limited by differences in source, method of collection/sampling, and validity of the measures. The CDI is a tool used globally to assess countries’ performance on child health, education, and nutrition [58]. Countries are ranked according to their scores based on a child’s risk of dying before his/her fifth birthday, of not enrolling in school, and of being underweight [58]. The CDI is measured on a scale of 0 to 100 with a higher number suggesting children are worse off [58]. To consider longer-term outcomes of child care and ECE, the educational achievement of 15 year-olds in reading, math and scientific literacy were reviewed. The OECD conducted a cross-sectional survey of 250,000 students in 41 countries [45]. Students were given a 2-hour test designed by a group of international experts that measured ability in literacy, numeracy, and science as applied to the management of everyday life [45]. Students were measured on a numerical scale with the OECD average being around 500 [59]. Equivalent data for Cuba were not available.

## Results

### Comparison of countries and redistributive policies

Table 1 shows demographic, economic, inequality, and social support information for the included countries. With the exception of Cuba, which is a communist country, all included countries have democratically-elected governments. All countries, with the exception of the United States, also provide free health insurance for children. All countries had a similar GDP per head ($39,000-$42,000) with the exception of Cuba which was significantly lower ($9,900) [9,11]. Sweden had the highest income distribution and lowest measured inequality (Gini coefficient 0.26, IHDI 0.851) and the United States had the lowest income distribution and highest measured inequality (Gini coefficient 0.38, IHDI 0.771) [11,15]. The Gini coefficient for Cuba was included for interest, but was not considered in the analysis as the data source was unknown, so it may not be comparable to OECD Gini coefficient calculations [14]. Additionally, for Cuba, only unadjusted HDI information was available, so it was not directly comparable with other countries. A similar pattern was seen for general fiscal support of families with Sweden contributing the most (3.4%) and the United States the least (0.7%) [11]. We were unable to find equivalent data for Cuba. Expenditures on child care and preschool education as a percentage of GDP ranged from 0.2% (Canada) to 1.1% (Sweden) [60]. No equivalent data were available for Cuba.

**Table 1:** Demographic, economic, inequality, and social support in Sweden, the Netherlands, Canada, the United States, and Cuba

	**Sweden**	**Netherlands**	**Canada**	**United States**	**Cuba**
**Political tradition** (1950–2000) [4]	Social democratic parties	Christian democratic parties or conservative parties in Judeo-Christian tradition	Liberal parties or conservative parties of a liberal persuasion	Liberal parties or conservative parties of a liberal persuasion	Conservative dictatorship
**Population, 2011** (millions) [11,12]	9.4	16.7	33.9	313.2	11.3
**Population density** (people per square kilometer of land), 2010 [12]	22.9	492.6	3.8	33.8	105.8
**Immigrants** (foreign born population), 2009 [11,12]	14.4%	11.1%	19.6%	12.7%	0.1%
**GDP** (output approach, USD, current prices and PPP*), 2010 (billions) [9,11]	$369.0	$701.0	$1,332.6	$14,447.1	$114.1
**GDP per capita** (US dollars, current prices and PPP*), 2010 [9,11]	$39,345	$42,218	$39,049	$46,587	$9,900
**Gini coefficient level** late 2000s, 2010 [11,14]	0.26	0.29	0.32	0.38	0.5**
**IHDI** (2011) [15]	0.851	0.846	0.829	0.771	0.776***
**Tax revenue** (taxes on income, profits and capital gains as a % of GDP), 2009 [9,11]	46.7%	38.2%	32.0%	34.3%	75.8%
**Public expenditures on family as a % of GDP,** 2007 [11]					
Cash benefits	1.5%	0.6%	0.8%	0.1%	No equivalent data available
Benefits in kind	1.9%	1.4%	0.2%	0.6%	
Total	3.4%	2.0%	1.0%	0.7%	
**Public expenditure on child care and early education services as a % of GDP**, 2007 [60]	1.1%	0.7%	0.2%	0.4%	No equivalent data available
**Health insurance funding**	Government	Private, children have free insurance	Government	Private with some government	Government

*Purchasing power parity.

**Interpret with caution as data source is not verifiable.

***Unadjusted for inequality.

### Prenatal care

Table 2 shows the policies and outcomes for prenatal care. All countries used midwives and/or physicians for prenatal, intra-partum and post-partum care. However, the degree to which different countries utilized these provider groups varied. For example, the Netherlands almost exclusively used midwives for prenatal care; whereas the percentage of physician-providers in Canada was much higher (92%) [19,61]. In the Netherlands and Sweden, where midwives provided the majority of prenatal care, the c-section rate was lowest (15.4% and 17.2%, respectively) [23,26]. The c-section rate was highest in the United States (32.9%) [24].

**Table 2:** Policies and outcomes for prenatal care

**Policy**	**Sweden**	**Netherlands**	**Canada**	**United States**	**Cuba**
Primary providers of prenatal care [19-22]	Midwives and obstetricians	Midwives	Obstetricians and family physicians	Obstetricians	Polyclinics (wide range of professionals available)
**Outcome**					
Maternal smoking rate (Canada 2007, Sweden and US 2009) [23-25,62]	6.9%	7.6%	10%	12.8%	No equivalent data available
C-section rate (Canada 2006,all others 2009) [19,20,23,24,26]	17.2%	15.4%	26.3%	32.9%	28.5%
Infant mortality rate (2005) (per 1000 live births) [11,12]	2.4	4.9	5.3	6.8	5.3
Low birth weight rate (<2500 g) (2003–2005) [11,12]	4.2%	6.2%	5.9%	8.1%	5.4%

The infant mortality rate ranged from 2.4 per 1000 (Sweden) to 6.8 per 1000 (United States) [63,64]. Sweden’s rate was significantly lower than the next lowest rate of 4.9 per 1000 (the Netherlands) and more than half the rate of Canada, the United States, and Cuba. Low birth weight rates (<2500 g) ranged from 4.2% in Sweden to 8.1% in the United States [20,65,66].

### Maternal leave

Table 3 shows the policies and outcomes for maternal leave. The maternal leave allowance ranged from 12 weeks in the United States to 68 weeks in Sweden [33,35]. The United States was the only country where maternal leave was not paid [35]. The included countries showed breastfeeding initiation rates from 70% (Cuba) to 97.6% (Sweden) [11,38]. The Netherlands, United States, and Canada all had breastfeeding initiation rates below 85% [11,37]. Exclusive breastfeeding at 6 months showed a significant decrease from initiation for all countries with a range of 10.4% in Sweden to 26% in Cuba [23,38]. Interestingly, Cuba had the lowest initiation rates, but best maintenance of exclusive breastfeeding at 6 months. The Netherlands also showed a comparatively high 6-month rate of 18%, whereas all of the remaining countries were below 15% [19,39].

**Table 3:** Policies and outcomes for maternal leave

**Policies**	**Sweden**	**Netherlands**	**Canada**	**United States**	**Cuba**
Maternal leave allowance [32-36]	480 days (~68 weeks). Might be used by the mother, the father, or mixed (most common)	16 weeks (mandatory 4 weeks prior to due date)	15 weeks	12 weeks	1 year
Maternal leave pay [32-36]	78% of income for 390 days Min $3 USD/day Max $130 USD/day	Full salary (no max payment)	55% of a woman’s average insured earnings up to a yearly max of $44,900 if worked 600 insured hours in the 52 weeks prior to delivery	Unpaid	Full salary for 18 weeks (6 weeks before birth and 12 weeks after)
Parental leave [32-36]	60 days (10 days paid leave at birth of child); either parent may take unpaid leave at 25% until the child is 8 years	Additional unpaid leave can be taken by either or both parents after delivery based on hours worked in a week until the child is 8 years	May be used by one parent or shared, but cannot exceed a combined max of 35 week; max payment is $485 per week	Unpaid	Additional 40 weeks leave at 60% pay may be taken by either parent
**Outcomes**					
Initiate breastfeeding (having ever breastfed) (2008, Netherlands 2010, Cuba 2006–2010) [11,37,38]	97.6%	75.0%	84.5%	74.2%	70%
Exclusive breastfeeding at 6 months (Sweden 2009, Netherlands 2010, Canada 2006, Cuba 2006–2010, US 2011) [19,23,37-39]	10.4%	18%	14.4%	14.8%	26%

### Child health care

Table 4 shows the policies and outcomes for child health care. Recommended preventive child visits (i.e., well baby visits) differed markedly in number and costs between the countries from 6 in the first 18 months in Canada to 20 visits during the same time period in Sweden. In Cuba there was a community-based, family-centered program that integrated health and education services into a single system, prioritising health, learning, behaviour, and life trajectories during prenatal life, infancy, childhood, and adolescence. In this program (*Educa a Tu Hijo*) children received between 104 and 208 stimulation and development monitoring sessions up to the age of 2 years [41]. Only Sweden and the Netherlands systematically tracked their service coverage rate of all preventive child health care visits. The under 5 years mortality rate showed a similar pattern with Sweden having the lowest mortality (3 per 1000 live births) and the United States the highest (8 per 1000 live births) [44]. Most countries were doing well in achieving high primary series vaccination uptake rates; Sweden, the Netherlands, the United States, and Cuba were all above 92% for the primary series of diphtheria, tetanus, pertussis, polio, haemophilius influenza B, and measles [44]. However, Canada has fallen to 80% for some vaccines [44].

**Table 4:** Policy and outcomes for child health care

**Policy**	**Sweden**	**Netherlands**	**Canada**	**United States**	**Cuba**
Acute care	General practitioners, paediatricians	Family physicians, paediatricians	Family physicians, paediatricians, nurse practitioners, emergency physicians	Family physicians, paediatricians, nurse practitioners, emergency physicians	Polyclinics
Preventive care	Public health nurses, general practitioners, paediatricians	Public health physicians and specialized nurses	Family physicians, paediatricians, nurse practitioners, public health nurses	Paediatricians, family physicians, nurse practitioners	Polyclinics
Number of recommended preventive care visits and approximate cost per child per year (USD) [41]	20 visits in the first 18 months ($275-1000)	10 visits in the first 18 months ($150)	6 recommended and 4 optional in the first 18 months ($140)	8 recommended visits in the first 18 months (costs vary based on health insurance plan co-payments and whether preventive visits are included in the deductible)	Children receive between 104 and 208 stimulation and development monitoring sessions up to the age of 2 yearsNo data on costs
**Outcomes**					
Service coverage rate preventive visits (Sweden 2008; Netherlands 2007) [42,43]	99%	95%	No equivalent data	No equivalent data	No equivalent data
Under 5 years mortality per 1000 live births (2010) [44]	3	4	6	8	6
Vaccination coverage (2010) [44]					
DPT1	99%	99%	92%	99%	98%
DPT3	98%	97%	80%	95%	96%
Polio3	98%	97%	80%	93%	99%
Measles	96%	96%	93%	92%	99%
Hib3	98%	97%	80%	93%	96%

### Child care and early childhood education

Table 5 shows the policies and outcomes for child care and ECE. Childcare and ECE programs and services varied markedly between and within countries; however some common themes emerged, including child day care beginning at birth to 6 months of age and typically continuing until school age, pre-school programs starting at 1–3 years, and compulsory school beginning at 5–7 years. All of the countries had variations on 3 main types of child care and ECE services, including daycare centres, preschools, and parent–child programs, although these also differed significantly between provinces in countries like Canada [67]. Additionally, very little was known about the quality of different ECE services across the countries. Canada had the best CDI at 0.74 and the United States the worst at 2.86 [58]. Despite the investment in early child education in many countries, systematic data collection on readiness at school entry was lacking. Canada and the United States collected some school readiness data, such as with the EDI, but data collection was patchy and not analyzed at a country-level. A follow-up of “*Educa a Tu Hijo*”, a Cuban program in which all pregnant woman have at least 12 prenatal medical checks and deliver in a maternity clinic or specialised health center, [2] showed that only 13% of participating children reached school age with unsatisfactory development in key domains (motor skills, cognition, social-personal, and personal hygiene) [2]. Looking at outcomes in 15 year-olds for reading, mathematics, and science literacy, where the OECD average was 500, all of the countries except for the United States scored above 500 [45]. Data were not available for Cuba.

**Table 5:** Policies and outcomes for child care and early childhood education

**Policy**	**Sweden**	**Netherlands**	**Canada**	**United States**	**Cuba**
Child care centres (formal or family-based) [42,46-49]					
Age	1-5 years	3 mos-4 years	Birth-6 years	Birth-6 years	6 mos-5 years
Hours of operation	Working days	Working days	Working days	Working days	Working days
Parental contribution	Mainly tax funded with some income-based parent fees	44%	Vary widely	Vary widely	Many free, although some up to 100%
Government contribution	Income-based (up to 100%)	Income- based (3.5-100%)	Vary widely	Vary widely	Up to 100%
Pre-school [50,51]					
Age	1-5 years	2-4 years	2-6 years	3-5 years	5 years
Hours of operation	Work days	3 x ½ day/week	2-3 hours/day	Varies	Twice weekly
Financing	Mainly tax-funded, parent fees based on income and number of children	Municipally-funded, may charge a fee to parents	Tax-funded and/or parent fees	Tax-funded and/or parent fees	Government-funded
Kindergarten (varies by state or province in Canada and the United States)					
Age may start	6 years	4 years	4 or 5 years	5 years	4-6 years
Compulsory age of start	7 years	5 years	6 years	5 or 6 years	7 years (Grade 1)
School readiness [2,52-55]	No equivalent data	No equivalent data	EDI (% vulnerable) 25% Ontario, 31% British Columbia, 29.1% Manitoba (not ready 1 or more domains for all)	21 states have no statewide readiness assessments; 6 states publish school readiness data; instruments vary e.g., 17% vulnerable on MMSR* in Maryland	13% unsatisfactory development in key domains (motor skills, cognition, social-personal and personal hygiene)
CDI 2005-2010 [58]	1.85	0.93	0.74	2.86	2.27
Reading literacy achievement aged 15 (score) (2003) [45]	514	513	528	495	No equivalent data
Mathematics literacy achievement aged 15 (score) (2003) [45]	509	538	532	483	No equivalent data
Science literacy achievement aged 15 (score) (2003) [45]	506	524	519	491	No equivalent data

*MMSR is the Maryland Model for School Readiness.

## Discussion

This paper compared four social determinants of early child development in five countries with varying redistributive policies. Sweden, the country with the greatest redistributive policies overall showed the best early child health and development outcomes. Countries with the least redistributive policies, like the United States, generally performed poorly in comparison. Although a comparative analysis cannot claim causation, our analysis suggests that redistributive policies aimed at reducing social inequalities are associated with a positive influence on the social determinants of early child development.

In comparing prenatal care services and outcomes, a decreasing trend in infant mortality rate and low birth weight rate was observed with an increase in redistributive policies. Cuba was an exception with an infant mortality rate comparable to Canada and a low birth weight rate second to Sweden. A possible explanation could be the high enrollment in comprehensive programs, such as “*Educa a Tu Hijo*” (discussed above). A higher c-section rate was observed in countries with less redistributive policies. However, this could also be explained by differences in service providers (i.e., more use of midwifery in Sweden and the Netherlands) or the fact that home deliveries in the Netherlands are popular as is the view of pregnancy and delivery as physiological events which should not be unnecessarily medicalised [68].

Generous redistributive policies were associated with higher maternal leave allowance and pay. Sweden had the highest breastfeeding initiation rate; however the exclusive breastfeeding rate at 6 months was the highest in Cuba and the Netherlands. Recent studies have revealed varied sociodemographic, biomedical, and psychosocial determinants of breastfeeding duration and exclusivity, which may account for these discrepancies [69].

In the comparison of child health care services, more preventive visits were recommended in countries with greater redistributive policies. Again, Cuba was an outlier with its high number of combined child health and development stimulation visits. No clear association was seen between immunization uptake rates and redistributive policies. This correlation may have been masked because of the small spread in values between the countries or an approach to vaccination policy which occurs irrespective of redistributive policies. A remarkable fact is that only Sweden and the Netherlands keep track of the coverage rate for preventive visits. A high-quality surveillance and monitoring system is critical to determining what support systems are needed for children [57].

In the analysis of child care and ECE, the lack of uniform measures of early child development outcomes was apparent. As stated by the American Academy of Pediatrics, school readiness is an important outcome for community-based development programs [70]. With the development and implementation of the EDI, Canada is one of the world leaders in measuring the outcomes of early child development with a population-based survey approach [57]. By expanding and enhancing measures of early child development, countries can more quickly address this important need and evaluate the impact of interventions on population-level outcomes [57].

### Strengths

We used a previously established method of categorizing countries by redistributive political traditions. This allowed for comparison between multiple countries in all of the categories, while keeping the number of countries manageable to allow for meaningful comparisons. Where data were available, they were generally robust and collected consistently across countries (e.g., immunization rates, infant mortality).

### Limitations

Global databases, such as OECD.Stat and World Data Bank rely on data collected by national statistics. Although these large databases have made increasing strides to standardize indicator definitions, data sources, and collection methodology, inherently, there will be differences between some countries that limit comparability. Additionally, not all policies and outcomes of interest were collected through standardized, country-level sources, making comparability more challenging for some indicators. Due to these limitations, we attempted to choose indicators with objective or widely recognized definitions. Additionally, some data points were missing as statistics were either not publicly available or not collected. Although this was most readily apparent for Cuba, we elected to include the data available, with cautionary notes on interpretation, as Cuba’s successes in some areas of early child health and development may serve as case studies for other resource-constrained settings.

Early child health and development is impacted by a web of different social, cultural, economic, genetic, political, and environmental conditions. While redistributive policies and indices, such as the Gini coefficient and IHDI, attempt to capture the complexity of this web, the categorization is inherently incomplete.

Another limitation is that we only included one country from each different political tradition in our analysis. However, if we examine UNICEF’s latest report about early childhood services, the same ranking is observed as in our study with the social democratic countries having the best and the liberal countries the worst early childhood outcomes [10]. UNICEF’s report proposed 10 internationally applicable benchmarks for early childhood care and education [10]. The report card scores ranged from 1–10, a 10 indicated that all suggested standards were met. The social democratic countries (Sweden, Denmark, Finland and Norway) all scored 8 or higher; the Christian democratic countries (the Netherlands, Germany and Belgium) scored between 4 and 6; and the liberal countries (Canada, Ireland, and US) scored the lowest, between 1 and 3. An exception was the United Kingdom which also has a liberal government and scored a 5, this is possibly explained by the “Sure Start Program” which offered full day care for children, good quality teacher input to lead the development of learning within the centre, and child and family health services for disadvantaged groups [2]. This underscores the statement of Navarro *et al.* that there is a great need for further research to establish the interactions between politics, policy, and health outcomes [4].

As discussed previously, a comparative analysis allowed for correlation, but not causation. In the east Asian welfare states (South Korea, Japan, Taiwan, Singapore, and Hong Kong), child health seems to be as good as in Scandinavia [71]. The east Asian welfare states are characterized by low social, educational, and health expenditures. Thus, the findings in our study might not be generalizable to countries outside the western tradition.

### Future directions

In order to better understand and continue to improve early child health and development, policy makers should consider the following:

•Establish mechanisms to systematically collect, analyze, and respond to early child health and development indicators. This information is essential to ongoing monitoring and improvement of the early child health and social systems;

•Promote redistributive policies aimed at reducing social inequality;

•Use a “health in all” approach to policy development, so that the health and implications of economic and social policies are understood and considered.

## Conclusions

We hope this paper will inform and encourage further discussion between health care professionals, child development specialists, and policy makers. In these economically challenged times, the need for supportive social and health policies for children and families are crucial to improve life course trajectories, not only for individual children, but also for society and human development globally.

## Abbreviations

c-section: Caesarean section; CDI: Child development index; ECD: Early child development; ECE: Early childhood education; EDI: Early development instrument; GDP: Gross domestic product; IHDI: Inequality-adjusted human development index; MMSR: Maryland model for school readiness; OECD: Organisation for economic cooperation and development; PPP: Purchasing power parity; USD: United States dollar.

## Competing interests

The authors declare that they have no competing interests.

## Pre-publication history

The pre-publication history for this paper can be accessed here:

http://www.biomedcentral.com/1471-2458/13/1049/prepub
